# A solvable model for the diffusion and reaction of neurotransmitters in a synaptic junction

**DOI:** 10.1186/2046-1682-4-5

**Published:** 2011-03-02

**Authors:** Jorge L Barreda, Huan-Xiang Zhou

**Affiliations:** 1 Department of Physics and Institute of Molecular Biophysics, Tallahassee, Florida 32306, USA

## Abstract

**Background:**

The diffusion and reaction of the transmitter acetylcholine in neuromuscular junctions and the diffusion and binding of Ca^2+ ^in the dyadic clefts of ventricular myocytes have been extensively modeled by Monte Carlo simulations and by finite-difference and finite-element solutions. However, an analytical solution that can serve as a benchmark for testing these numerical methods has been lacking.

**Result:**

Here we present an analytical solution to a model for the diffusion and reaction of acetylcholine in a neuromuscular junction and for the diffusion and binding of Ca^2+ ^in a dyadic cleft. Our model is similar to those previously solved numerically and our results are also qualitatively similar.

**Conclusion:**

The analytical solution provides a unique benchmark for testing numerical methods and potentially provides a new avenue for modeling biochemical transport.

## 1. Background

In intercellular and intracellular spaces, passive transport of biomolecules is a common phenomenon. Because such processes are difficult to probe directly by experiments, numerical modeling is increasingly used to gain insight. Two processes that have been extensively modeled are the diffusion and reaction of the transmitter acetylcholine in a neuromuscular junction [1-6] and the diffusion and binding of Ca^2+ ^in the dyadic cleft of a ventricular myocyte [7,8]. In contrast to previous numerical approaches, here we present an analytical solution of a model for the diffusion and reaction of acetylcholine in a synaptic cleft (or Ca^2+ ^in a dyadic cleft). Our model is similar to those previously solved numerically; hence our analytical solution potentially provides a new avenue for modeling biochemical transport. More importantly, an analytical solution provides a unique benchmark for testing numerical methods. Such a solution has been lacking up to now; the present work fills this gap.

**Figure 1:**
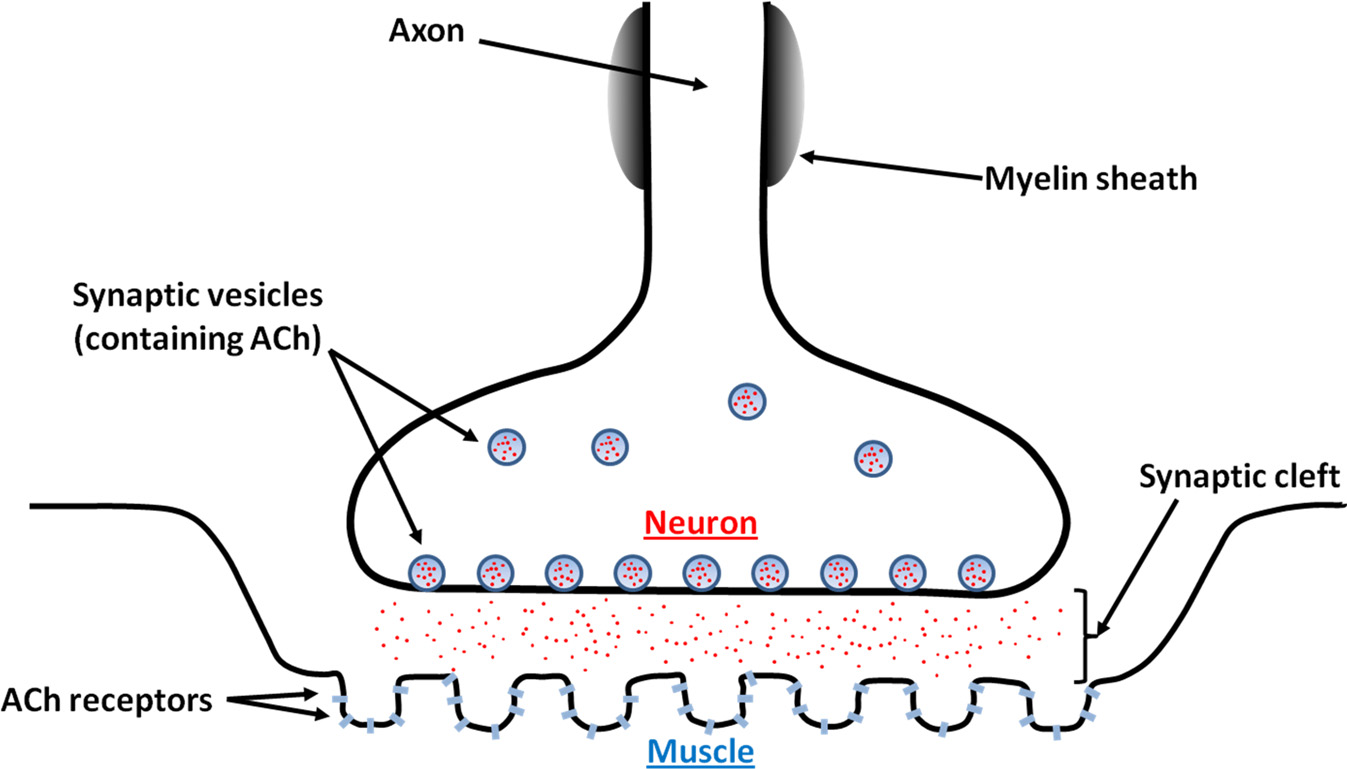
**Illustration of a neuromuscular junction**. Presented are the key players in the diffusion and reaction of the neurotransmitter, acetylcholine (ACh).

Neuromuscular junction refers to the cleft between a motor neuron and a muscle fiber. As illustrated in Figure 1, the neuronal signal for muscle contraction is mediated by acetylcholine. These neurotransmitter molecules are initially inside vesicles located in the pre-synaptic axon terminal. When an action potential reaches the axon terminal, the vesicles release acetylcholine molecules into the synaptic cleft. These molecules then diffuse toward the post-synaptic membrane and bind to acetylcholine receptors in the membrane. Acetylcholine binding activates these ligand-gated ion channels, allowing Na^+ ^to flow in and generating an action potential along the muscle fiber. Finally excess acetylcholine molecules around the post-synaptic membrane are broken down by acetylcholinesterase to prevent continued activation of acetylcholine receptors.

**Figure 2:**
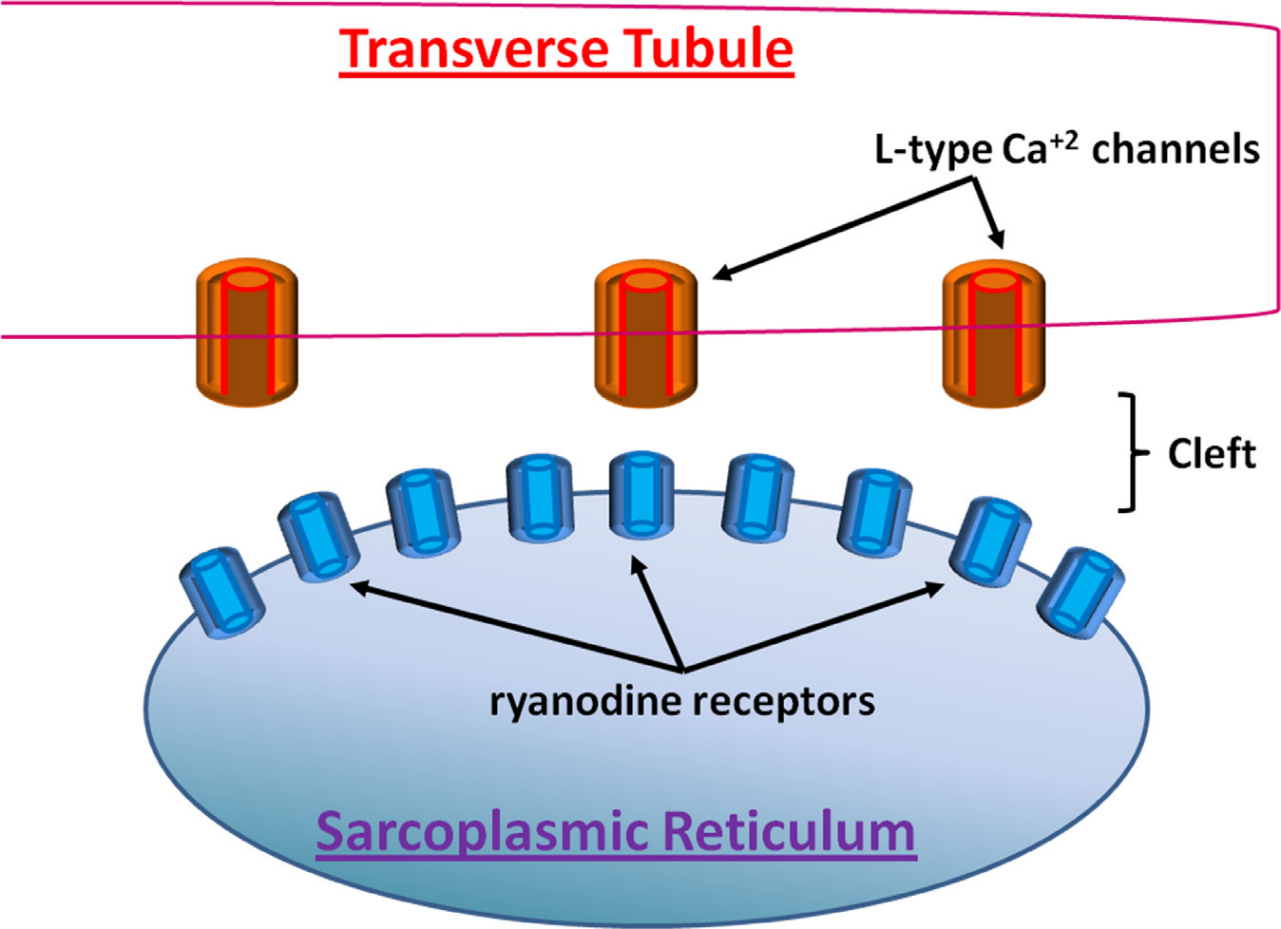
**Illustration of a dyadic cleft**. Presented are the key players in the diffusion and binding of Ca^2+^.

A related system is a dyadic cleft, which spans the gap between the cell membrane in a transverse tubule and the membrane of a sarcoplasmic reticulum. As Figure 2 illustrates, Ca^2+ ^can enter the cell through L-type Ca^2+ ^channels on the cell membrane in response to the arrival of an action potential. The ions then diffuse to reach and activate ryaonodine receptors in the membrane of the sarcoplasmic reticulum. The activated ryaonodine receptors release Ca^2+ ^from the sarcoplasmic reticulum, which ultimately lead to muscle contraction.

**Figure 3:**
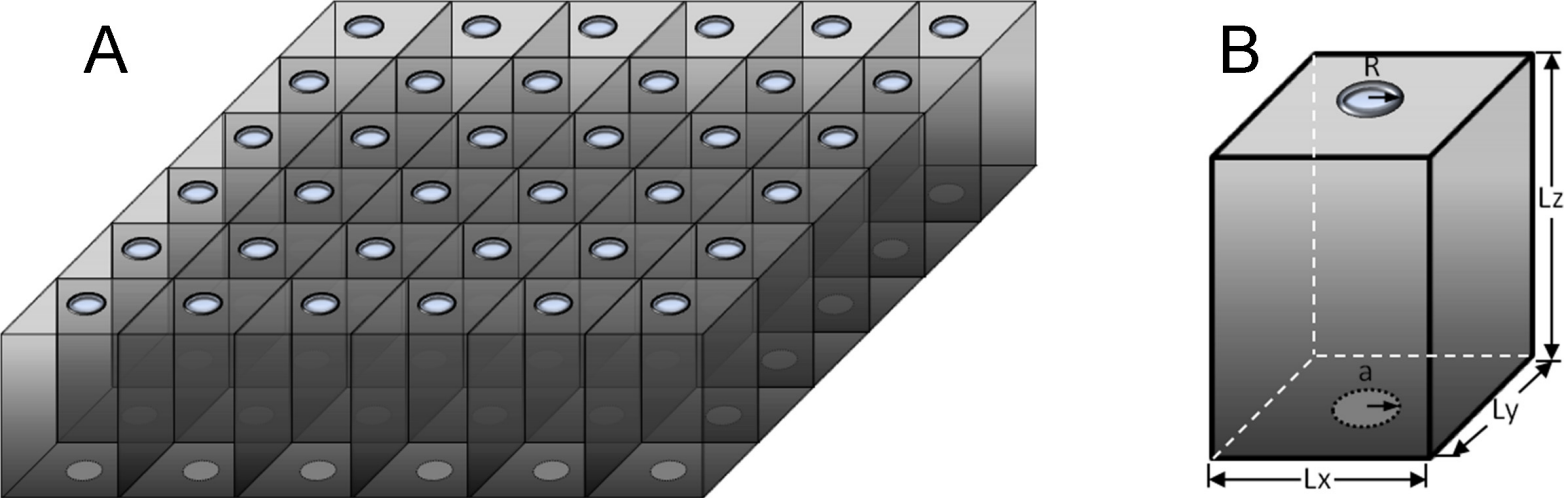
**Our model for both a neuromuscular junction and a dyadic cleft**. Panel A: The pre-cleft membrane (top) contains a periodic array of disks for the influx of ligands; the post-cleft membrane (bottom) contains a periodic array of absorbing disks representing receptors. Panel B: The dimensions of a unit cell.

Here we propose a simple but not unrealistic model for the diffusion and reaction of acetylcholine in a neuromuscular junction. The model also applies to the diffusion and binding of Ca^2+ ^in a dyadic cleft. We are able to find an analytical solution for this model. For convenience we describe our model in the language of neuromuscular junction. As shown in Figure 3, we model the synaptic cleft as the space between two infinite, parallel planar membranes. In the pre-synaptic membrane, there is a periodic array of circular openings, via which acetylcholine molecules enter the cleft. In the post-synaptic membrane, there is a periodic array of circular disks, where the acetylcholine molecules are absorbed. The quantity of interest is the total flux, *J*(*t*), at time *t *of acetylcholine molecules across the post-synaptic membrane. This model allows us to use periodic boundary conditions in the transverse direction. Our results are qualitatively similar to those obtained previously by numerical solutions [1-7]. More realistic ingredients can be added to our model and still permit analytical solutions.

## 2. Methods

We set up a coordinate system such that the *x *and *y *axes are parallel to the pre- and post-synaptic membranes. The synaptic junction has depth *L_z_*. The junction is periodic in the *x *and *y *directions, with periodicities of *L_x _*and *L_y_*, respectively. In each "unit cell", a synaptic vesicle bursts at time *t *= 0, releasing the neurotransmitters into the cleft. We model the release of the neurotransmitters as a transient flux, *u*(*t*), that is confined to a circular opening with radius *R*. We place the synaptic vesicle at the center of the pre-synaptic face of the unit cell. After diffusing to the post-synaptic membrane, neurotransmitters are absorbed by a circular disk in each unit cell, with radius *a*. We place this "sink" also at the center of the post-synaptic face of the unit cell. The exact shapes and locations of the pre-synaptic opening and the post-synaptic sink are not essential for the analytical solution of our model. The quantity of interest is the total flux, *J*(*t*), through the post-synaptic face of each unit cell.

We place the origin of the Cartesian coordinate system at the center of the pre-synaptic face, with the *z *axis pointing toward the post-synaptic face. The concentration of neurotransmitters at position **r **and at time *t *is *C*(**r**, *t*). Within the synaptic junction, it is governed by the diffusion equation,

(1) $$\frac{\partial C(r,t)}{\partial t}=D\nabla^{2}C(r,t)$$

where *D *is the diffusion constant. The initial condition is

(2) C(r,0)=0

The boundary condition at *z *= 0 is

(3a) $$D\frac{\partial C(r,t)}{\partial z}=u(t)\;\text{when}\;\rho <R$$

(3b) $$D\frac{\partial C(r,t)}{\partial z}=0\;\text{when}\;\rho >R$$

where *ρ *= (*x*^2 ^+ *y*^2^)^1/2 ^is the distance to the *z *axis. The boundary condition at *z *= *L_z _*is

(4a) C(r,t)=0 when ρ <a

(4b) $$D\frac{\partial C(r,t)}{\partial z}=0\;\text{when}\;\rho >a$$

We solve the problem in Lapace space. For a function *f*(*t*) of *t*, we denote the Laplace transform as $\hat{f}(s)\equiv \int_{0}^{\infty }dte^{-st}f(t)$. The Laplace transform of Eq. (1), using the initial condition of Eq. (2), is

(5) $$s\hat{C}(r,s)=D\nabla^{2}\hat{C}(r,s)$$

The solution appropriate for the periodic boundary conditions in the *x *and *y *directions has the form

(6) $$\hat{C}(r,s)=\sum_{l,m=0}^{\infty }\left[\alpha_{lm}(s)e^{\gamma_{lm}(s)z}+\beta_{lm}(s)e^{-\gamma_{lm}(s)z}\right]\cos(2l\pi x/L_{x})\cos(2m\pi y/L_{y})$$

where

(7) $$\gamma_{lm}(s)={\left[s/D+{\left(2l\pi /L_{x}\right)}^{2}+{\left(2m\pi y/L_{y}\right)}^{2}\right]}^{1/2}$$

The coefficients *α_lm_*(*s*) and *β_lm_*(*s*) are to be determined from boundary conditions.

To make use of the boundary condition of Eq. (3), we need the 2-dimensional cosine transform of a function in *x *and *y *that has value 1 when *ρ *<*R *and value 0 when *ρ *>*R*. The coefficient of the cos(2*lπ*/*L_x_*)cos(2*mπ*/*L_y_*) term is

(8) $$q_{lm}=\frac{4\varepsilon_{l}\varepsilon_{m}}{L_{x}L_{y}}\iint_{\rho <R}dxdy\cos(2l\pi x/L_{x})\cos(2m\pi y/L_{y})$$

where *ε*_0 _= 1/2 and *ε_l _*= 1 for *l *> 0. For later reference, we note that the function having value 1 when *ρ *<*a *and value 0 when *ρ *>*a *has the following coefficients in its 2-dimensional cosine transform:

(9) $$p_{lm}=\frac{4\varepsilon_{l}\varepsilon_{m}}{L_{x}L_{y}}\iint_{\rho <a}dxdy\cos(2l\pi x/L_{x})\cos(2m\pi y/L_{y})$$

Applying the boundary condition of Eq. (3), we find

(10) $$D\gamma_{lm}(s)\left[\alpha_{lm}(s)-\beta_{lm}(s)\right]=\hat{u}(s)q_{lm}$$

For the boundary condition of Eq. (4) at *z *= *L_z_*, we use the constant-flux approximation [9]:

(11a) $$D\frac{\partial \hat{C}(r,s)}{\partial z}=Q(s)\;\text{when}\;\rho <a$$

(11b) $$D\frac{\partial \hat{C}(r,s)}{\partial z}=0\;\text{when}\;\rho >a$$

where the quantity *Q*(*s*) is to be determined by the condition

(12) $$<\hat{C}(r,s)>\equiv {\left(\pi a^{2}\right)}^{-1}\iint_{\rho <a}dxdy\hat{C}(r,s)=0$$

Equation (11) leads to

(13) $$D\gamma_{lm}(s)\left[\alpha_{lm}(s)e^{\gamma_{lm}(s)L_{z}}-\beta_{lm}(s)e^{-\gamma_{lm}(s)L_{z}}\right]=Q(s)p_{lm}$$

With Eqs. (10) and (13), we solve for coefficients *α_lm_*(*s*) and *β_lm_*(*s*). The results are

(14a) $$\alpha_{lm}e^{\gamma_{lm}(s)L_{z}}=\frac{1}{D\gamma_{lm}(s)}\frac{-q_{lm}\hat{u}(s)+p_{lm}Q(s)e^{\gamma_{lm}(s)L_{z}}}{e^{\gamma_{lm}(s)L_{z}}-e^{-\gamma_{lm}(s)L_{z}}}$$

(14b) $$\beta_{lm}e^{-\gamma_{lm}(s)L_{z}}=\frac{1}{D\gamma_{lm}(s)}\frac{-q_{lm}\hat{u}(s)+p_{lm}Q(s)e^{-\gamma_{lm}(s)L_{z}}}{e^{\gamma_{lm}(s)L_{z}}-e^{-\gamma_{lm}(s)L_{z}}}$$

Inserting Eq. (6) in Eq. (12) and using Eqs. (14) and (9), we find *Q*(*s*) as

(15) $$Q(s)=\hat{u}(s)\frac{\sum_{l,m=0}^{\infty }\frac{p_{lm}q_{lm}}{\varepsilon_{l}\varepsilon_{m}\gamma_{lm}(s)\sinh[\gamma_{lm}(s)L_{z}]}}{\sum_{l,m=0}^{\infty }\frac{p_{lm}{}^{2}\cosh[\gamma_{lm}(s)L_{z}]}{\varepsilon_{l}\varepsilon_{m}\gamma_{lm}(s)\sinh[\gamma_{lm}(s)L_{z}]}}$$

Finally the total flux through the sink on the post-synaptic face is

(16) $$\hat{J}(s)=\pi a^{2}Q(s)$$

The flux accumulated over all times,

(17a) $$I=\int_{0}^{\infty }dtJ(t)$$

is of interest. In our model,

(17b) $$I=\hat{J}(0)=\pi R^{2}\hat{u}(0)$$

which is independent of *a*. Equation (17b) is simply a consequence of ligand conservation, i.e., the total number of ligands released from the synaptic vesicle is the same as the total number of ligands absorbed by the receptors.

The analytical solution derived above can be implemented on any function *u*(*t*) modeling neurotransmitter release from the synaptic vesicle. We focused on an exponentially decaying function:

(18a) $$u(t)=e^{-t/t_{0}}$$

Its Laplace transform is

(18b) $$\hat{u}(s)=\frac{1}{s+1/t_{0}}$$

Our model then contains two parameters related to time: *D *and *t*_0_. For two sets of these parameters, e.g., *D*_1 _and *t*_01 _in one and *D*_2 _and *t*_02 _in the other, it can be shown that the corresponding response functions satisfy

(19) $$J_{1}(D_{2}t)=J_{2}(D_{1}t)\;\text{when}\;D_{1}t_{01}=D_{2}t_{02}$$

We now briefly describe the details of our implementation of the analytical solution. To calculate the *q_lm _*and *p_lm _*coefficients of Eqs. (8) and (9), we first carried out the integration over *y *analytically. The remaining integration over *x *was done numerically using the Gauss-Legendre quadrature with two points. The summations over *l *and *m *in Eq. (15) were truncated at *l *= *m *= 40. The Laplace transform of $\hat{J}(s)$ was inverted by the Stehfest algorithm [10]. A Fortran90 code for the implementation is available upon request.

## 3. Results

We now present some illustrative results. The parameters of our model are as follows: *L_x _*= *L_y _*= 500 nm; *L_z _*= 50 nm; *R *= 20 nm; *a *varied from 2.5 to 40 nm; *t*_0 _varied from 1 to 10 ms; and *D *varied in (0.4-4) × 10^5 ^nm^2^/ms.

**Figure 4:**
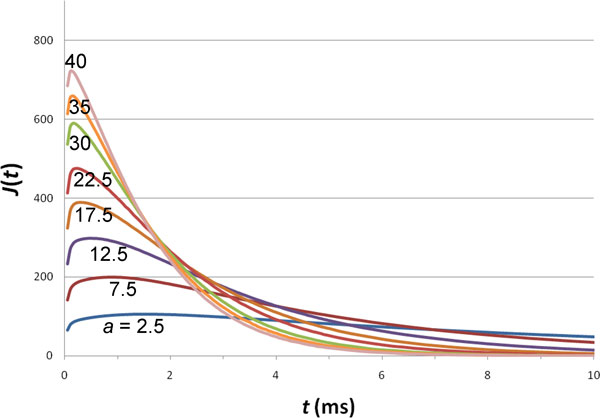
**Effect of the size of the absorbing disk on the response function**. The values of *a *in nm are shown in the figure. For all curves, *t*_0 _= 1 ms and *D *= 10^5 ^nm^2^/ms.

Our single absorbing disk is used to model ligand binding to multiple receptors. Increasing *a *thus mimics an increasing number, *N*_rec_, of receptors per unit cell. We expect *a *to increase linearly with increasing *N*_rec _when *N*_rec _is small; the increase in *a *then slows down at higher *N*_rec _(see Discussion). Figure 4 displays the dependence of the response function *J*(*t*) on *a *(and hence *N*_rec_). As *a *(i.e., *N*_rec_) increases, *J*(*t*) more and more quickly reaches a higher and higher maximum and decays faster and faster. This is the expected behavior. Moreover, according to Eqs. (17b) and (18b), the areas under the curves for different *a *values are all equal to *πR*^2^*t*_0_. Note also that all our *J*(*t*) curves have the familiar shape seen in previously numerical studies [1-7].

**Figure 5:**
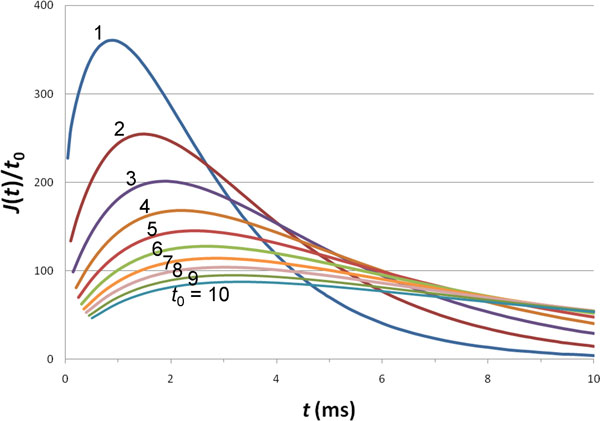
**Effect of the speed of ligand release on the response function**. Scaling of *J*(*t*) by *t*_0 _is to ensure that the same number of neurotransmitters is released for all the curves with different *t*_0 _values, which are shown in the figure in ms. *a *= 10 nm and *D *= 2 × 10^5 ^nm^2^/ms.

Figure 5 shows the change in the response function when the speed at which the neurotransmitters are released into the synaptic cleft is varied. To make a fair comparison, *J*(*t*) is scaled by *t*_0 _so that, effectively, the total number of neurotransmitters entering the synaptic cleft is fixed. It is clear that, as *t*_0 _increases (i.e., as the speed of neurotransmitter release decreases), the response function rises and decays more slowly. This behavior has previously been specifically modeled by Stiles et al. [1].

**Figure 6:**
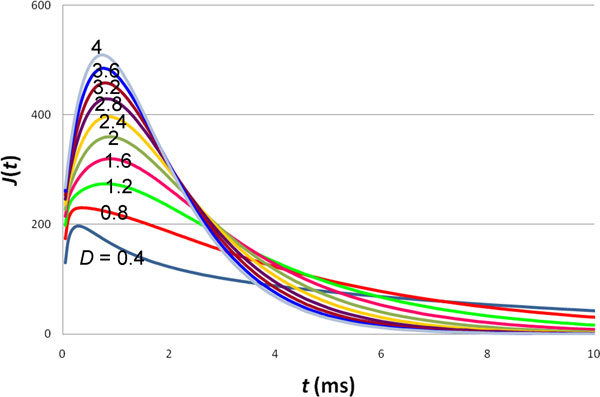
**Effect of the ligand diffusion constant on the response function**. The values of *D *in 10^5 ^nm^2^/ms are shown in the figure. For all curves, *a *= 10 nm and *t*_0 _= 1 ms.

There is some uncertainty on the diffusion constant of acetylcholine in neuromuscular junctions. In previous models [1-6], the value of *D *ranged from (0.25-6) × 10^5 ^nm^2^/ms. In Figure 6 we display the change in the response function when *D *is varied from (0.4-4) × 10^5 ^nm^2^/ms. As expected, when neurotransmitter diffusion slows down, the response function is also delayed. In our model, decreasing *D *has the same effect on the response function as increasing *t*_0 _[see Eq. (19)].

## 4. Discussion and Conclusion

We have presented an analytical solution to a model for the diffusion and reaction of acetylcholine in a neuromuscular junction. The model also applies to the diffusion and binding of Ca^2+ ^in a dyadic cleft. Our results are qualitatively similar to those obtained previously from models solved numerically [1-7].

Perhaps the greatest value of our analytical solution is that it provides a benchmark for testing numerical methods. Diffusion and reaction of ligands in intercellular and intracellular spaces have been modeled either on a particle description or a concentration description. The former type of models have been solved by Monte Carlo simulations [1,7], while the latter type of models have been solved by either finite-difference [2,6] or finite-element [3-5] methods. The two types of models have been shown to give equivalent results [8]. The level of realism of our model approaches those of the models solved numerically; hence our analytical solution will be able to serve as a good benchmark for the numerical methods.

Our model has room for increasing the level of realism and still allows for analytical solution. For example, we modeled ligand binding to receptors as absorbing. The binding can be modeled as partially absorbing if binding does not occur at every ligand-receptor encounter. The partial absorption condition takes the form

(20) $$D\frac{\partial C(r,t)}{\partial z}=\kappa C(r,t)\;\text{when}\;\rho <a$$

where the reactivity *κ *controls the degree of partial absorption. When *κ *= 0, the region *ρ *<*a *becomes reflecting and binding cannot occur. When *κ *→ ∞, the region *ρ *<*a *becomes fully absorbing and Eq. (20) reduces to the absorbing condition of Eq. (4a). Note that the bimolecular rate constant *k *for binding to the partially absorbing disk is given by [11]

(21) $$\frac{1}{k}=\frac{1}{4Da}+\frac{1}{\pi \kappa a^{2}}$$

One can thus parameterize *a *and *κ *by matching *k *with experimental data for the ligand-receptor binding rate constant.

Another simplification of our model is that a single absorbing disk is used to represent the receptors. We accounted for the presence of multiple receptors per unit cell by increasing the radius *a *of this disk. One can identify *a *by requiring that the bimolecular rate constant *k *calculated for the single absorbing disk is the same as that for the *N*_rec _receptors. For the single absorbing disk we have *k *= 4*Da *[see Eq. (21)]. If each of the *N*_rec _receptors is modeled as an absorbing disk with a small radius *a*_0_, then *k *≈ 4*N*_rec_*Da*_0 _when *N*_rec _is small [12]. Therefore *a *≈ *N*_rec_*a*_0 _for small *N*_rec_. As *N*_rec _increases, the rate constant *k *for the multiple receptors and correspondingly the radius *a *of the equivalent disk also increase, but the increase slows down as *N*_rec _becomes large [12,13]. Instead of using a single equivalent absorbing disk, analytical solution is actually still permitted when multiple receptors, each represented by a (partially) absorbing small disk, are present at arbitrary positions on the post-synaptic face. An alternative way to model the presence of multiple receptors is to assume that the whole post-synaptic face is partially absorbing, with an reactivity given by [12,13]

(22) $$\text{K}=\frac{N_{\text{rec}}k_{0}}{S(1-f_{\text{rec}})}$$

where *S *= *L_x_L_y _*is the area of the post-synaptic face, *f*_rec _= *N*_rec_*πa*_0_^2^/*S *is the fraction of the post-synaptic face that is covered by the receptors, and *k*_0 _is given by

(23) $$\frac{1}{k_{0}}=\frac{1}{4Da_{0}}+\frac{1}{\pi \kappa a_{0}{}^{2}(1-f_{\text{rec}})}$$

We have modeled ligand-receptor binding as irreversible. This is somewhat justified for modeling the neuromuscular junction, in which acetylcholinesterase can break down acetylcholine molecules newly released from the receptors. No such mechanism is present for Ca^2+ ^in the dyadic cleft. Reversible binding can be treated by appropriate boundary conditions [14] on the post-synaptic face. Another important detail is that both acetylcholine and ryanodine receptors have multiple binding sites for their ligands so that there are multiple ligand-occupation states for the receptors. Again, these can be treated by appropriate boundary conditions.

The geometries of some of the models previously solved numerically are more sophisticated than that of our model. In particular, secondary folds of the neuromuscular junction has been included in some of the previous models [1,3-5]. A formalism for treating ligand binding to a site buried in a narrow tunnel has been developed [15] and may be adopted for treating the narrow secondary folds in the neuromuscular junction. However, analytical solution requires idealized geometries; the kind of realistic geometries drawn from electron microscopy that can be handled by a finite-element method [4,5] is beyond the reach of analytical solution. Nevertheless, with all the new ingredients outlined above, analytical solution will potentially provide a new avenue for modeling biochemical transport.

## Authors' contributions

JLB implemented the analytical solution, did the calculations, and prepared the figures. HXZ derived the analytical solution and wrote the paper. Both authors read and approved the final manuscript.
